# Distributed Sensitivity and Critical Interference Power Analysis of Multi-Degree-of-Freedom Navigation Interference for Global Navigation Satellite System Array Antennas

**DOI:** 10.3390/s24020650

**Published:** 2024-01-19

**Authors:** Yuchen Jiang, Jun Fu, Bao Li, Pengfei Jiang

**Affiliations:** 1 School of Electrical Engineering, Naval University of Engineering, Wuhan 430033, China; jiangyuchen797@outlook.com (Y.J.); fjsd@21cn.com (J.F.); 2 College of Intelligent Science and Technology, National University of Defense Technology, Changsha 410073, China; hg_jpf@163.com

**Keywords:** multi-DOF, critical power, navigational interference, interference resolution

## Abstract

Current research on the interference of GNSS (Global Navigation Satellite System) array antennas focuses on the single interference effect and the improvement of interference hardware capability, while the multi-degree-of-freedom (DOF) interference model and mechanism remain to be fully studied. Aiming at this problem, this paper analyzes the preconditions for the definition of anti-jamming degrees of freedom and the characteristics of super-DOF interference through formula derivation and simulation. First, by analyzing the influence of the number of interfering signals on the angular resolution, the prerequisite of the definition of anti-interference degrees of freedom in the airspace is proposed. Second, the definition of anti-interference degrees of freedom is used to calculate the change rule of the critical power of the interference under different numbers of interfering signals. Finally, the influence of super-DOF interference on the array antenna is analyzed. The results show that the prerequisite for the anti-interference freedom of the array antenna is that the distribution interval of the interfering signal is greater than 15°, taking a four-array element uniform circular array antenna as an example. The critical interference power of the array antenna decreases by about 15 dB when the number of interfering signals exceeds the degrees of freedom of the array antenna’s interference immunity, provided that the interference resolution is satisfied. The conclusions of this paper give the critical power change rule of multi-DOF interference and the effect of super-DOF interference, as well as the prerequisites for the setting of interference signals, which can be used, for example, in the deployment of distributed interference sources and the development of anti-jamming algorithms.

## 1. Introduction

Satellite navigation systems are common and indispensable in the navigation and aviation fields, providing full-time, continuous, high-precision time, position, and speed information. As a satellite navigation signal is affected by factors such as the transmission distance in space and the ionosphere, the signal power is −130 dBm when it reaches the ground, and the weak signal power is susceptible to various types of interference [1]. A GNSS (Global Navigation Satellite System) array antenna receiver will see more intentional or unintentional interference, resulting in performance degradation, which has a great impact and can even become an important factor in determining victory or defeat [2].

Interference is categorized as follows.

Suppression interference is the launch of a certain bandwidth causing a GNSS receiver to not receive satellite signals normally. (1st) In narrowband interference, the bandwidth occupied by the interfering signal is much smaller than the bandwidth of the received signal, or else the absolute bandwidth of the interfering signal is very narrow, such as in monotone, single-frequency, or impulse interference. (2nd) In broadband interference, the bandwidth occupied by the interfering signal is larger than the bandwidth of the received signal, or else the absolute bandwidth of the interfering signal is very wide, such as in broadband AM, broadband FM, and frequency-sweeping interference. Usually, an interference bandwidth that is less than 1% of the entire signal bandwidth is considered narrowband interference, and it is considered broadband interference when it exceeds 10% [3]. Equipment for navigational uses, such as in the current Infauna systems, can suppress an adversary’s radio-electronic communications and various types of UAV navigation systems, operating in mountainous terrain up to a distance of 100 km [4].

Spoofing is the use of fake navigation signals sent to a receiver so that it obtains the wrong pseudo-range and thus calculates the wrong positional velocity information [5,6,7].

Distributed jamming has become a key technology [8]. With the continuous development of navigation jamming technology, the number of jamming sources usually exceeds the number of array elements of a GNSS antenna, resulting in the receiver’s inability to solve the effective position [9]. Subject to the space limitation of the piggyback platform, the number of array elements of L-band GNSS array antennas is usually four or seven, so it is easier to increase the number of interference sources than the number of array elements [10,11]. Many studies have been published on array antenna super-DOF interference suppression techniques, and some researchers have suggested that M-element GNSS antenna arrays can suppress up to M-1 interferences, but this is inaccurate, considering that the prerequisites are not limited to a specific range, while the signal types and parameters are not restricted [12]. Most beamforming and DOA (Direction of Arrival) estimation algorithms assume that the number of interfering signals is fewer than the number of antenna elements (M) [13]. Hence, research on array antenna degrees of freedom is gradually deepening, including super-DOF interference suppression performance that is more sensitive to the direction and distribution of the interference and studies the upper and lower bounds of super-DOF interference suppression performance, as well as the change rule in the azimuth, but gives no specific change rule of the distribution angle [14]. An array antenna receiver anti-jamming method based on antenna rotation was proposed [15], with strong foresight in the field of anti-jamming performance evaluation and anti-jamming algorithm development. Some researchers have proposed that if the number of interfering individuals is greater than or equal to the number of array elements, then the interference may not exceed the array’s degrees of freedom, and the existence of a special direction enables the antenna array to completely suppress super-DOF interference. However, this is specific to the interference deployment method and power requirements and cannot represent a generalized practical study. The results of the above studies may help suppress super-DOF interference, but there are limitations, such as algorithm complexity and hardware [16,17,18,19]. Currently, researchers are focusing on the study of multi-DOF anti-jamming performance without considering the characteristics and laws of multi-DOF jamming.

This paper starts from the perspective of spatial anti-interference. We first analyze the precondition that an M-element array can suppress at most M-1 interferences and give the specific spatial anti-interference angular resolution. Second, according to theoretical analysis and simulation, we propose that the array antenna’s weights do not converge in the case of super-DOF interference and give the critical power of single-frequency, wide-band FM and forwarding spoofing interference. The change rule is given.

**Figure 1:**
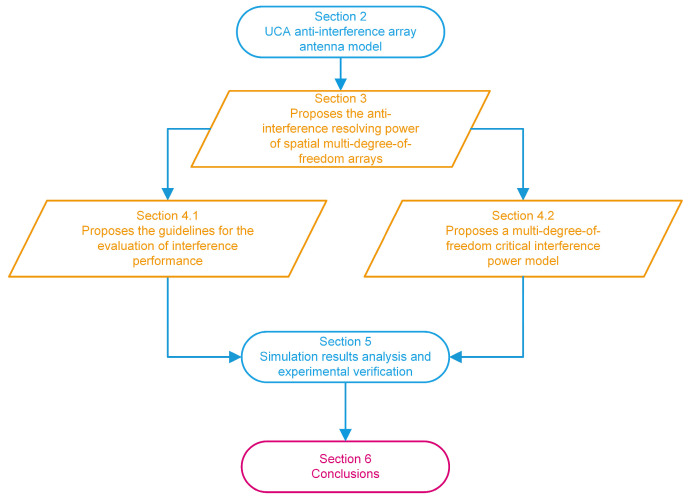
Block diagram of this work.

The remainder of this paper is structured as follows. The array signal reception model is established in Section 2. In Section 3, the preconditions for the definition of multi-DOF interference are proposed and illustrated by simulation. In Section 4, we propose the evaluation criterion of interference efficacy, based on a theoretical analysis, and establish the critical power model at the time of ultra-DOF interference. Section 5 analyzes our conclusions and verifies them by simulation and experiments. Figure 1 shows a structural block diagram of this work, whose orange parts identify our innovations.

Table 1 lists commonly used symbols.

## 2. Anti-Jamming Array Antenna Modeling

In a navigation system, the satellite signal in the propagation process of the physical environment is more complex. For this reason, we make the following assumptions when establishing the anti-jamming model of the array antenna:

(1) The size of each element is much smaller than the wavelength of the incident signal, which can be regarded as a point element at this time;
(2) The system noise is additive Gaussian white noise with mean zero and variance $\sigma^{2}$ , and the noise between the array elements, useful signal, and noise are independent of each other;
(3) The research content of this paper is the effect of a far-field interference signal on the array antenna, without considering the mutual coupling effect between the array elements and the channel inconsistency problem.

At present, the uniform circular array (*UCA*) and uniform linear array (*ULA*) structural forms are the most widely used. The *ULA* is characterized by a simple structure and convenience for DOA analysis in a one-dimensional direction, but its effect is poor in high-latitude analysis. Therefore, for analysis, we use the four-array *UCA*, with better directional characteristics and less influence of mutual coupling between the array elements, which can provide good analytical conditions for the study [20,21].

An M-element array antenna simultaneously receives navigation signals, noise signals $X_{N}$ , and interference signals $X_{J}$ . Let the signal received by the array antenna be

(1) $$X=X_{S}+X_{J}+X_{N},$$

where $X={\left[X_{1}\left(t\right)X_{2}\left(t\right)X_{3}\left(t\right)\cdots X_{N}\left(t\right)\right]}^{T}$ . The navigation signals $X_{S}$ can be represented as

(2) $$X_{S}=x_{s}=\sum_{l=1}^{L}p_{S_{l}}s_{l}\left(t\right)a_{S_{l}}\left(\theta_{l},\varphi_{l}\right)$$

where $p_{S}$ is the navigation signal power, $S_{l}$ is the time-domain navigation signal (TDNS), t is time, and $a_{S_{l}}$ is the direction vector of the satellite signal:

(3) $$a_{S_{l}}\left(\theta ,\varphi \right)={\left[1,e^{j\varphi_{l}\left(\theta ,\varphi \right)},\cdots ,e^{j\left(m-1\right)\varphi_{l}\left(\theta ,\varphi \right)}\right]}^{T}.$$

Then, the direction vector matrix of the whole navigation signal can be expressed as

(4) $$\begin{array}{c} A=\left[a_{1},a_{2},\cdots ,a_{L}\right]\\ =\left(\begin{array}{ccc} 1 & \ldots & 1\\ \vdots & \ddots & \vdots \\ e^{j\left(m-1\right)\varphi_{L}\left(\theta ,\varphi \right)} & \cdots & e^{j\left(m-1\right)\varphi_{L}\left(\theta ,\varphi \right)} \end{array}\right) \end{array}.$$

Interference signals are expressed in the same way as navigation signals. The noise signal $X_{N}$ can be expressed as

(5) $$X_{N}=n\left(t\right)={\left[n_{1}\left(t\right),n_{2}\left(t\right),\cdots ,n_{m}\left(t\right)\right]}^{T},$$

where $n\left(t\right)$ is additive Gaussian white noise with mean zero and variance $\sigma^{2}$ , m denotes the different array elements in the array antenna, and $n_{m}\left(t\right)$ denotes the input noise of each array element at the moment t , and

(6) $$E\left\{n_{i}\left(t\right)n_{j}^{k}\left(t\right)\right\}=\left\{\begin{array}{c} \sigma^{2},i=j\\ 0,i\neq j \end{array}\right..$$

Bringing the abovementioned model to a uniform circular array, assuming that the radius of the circle where the first element is located is R , the pitch angle is θ , and the direction angle is $\varphi_{l}$ , the direction unit vector of each element is

(7) $$a_{S_{m}}=\cos\varphi_{m}a_{S_{x}}+\sin\varphi_{m}a_{S_{y}}.$$

The unit vector in the direction of the field point is

(8) $$a_{r}=\sin\theta \cos\varphi_{m}a_{x}+\sin\theta \sin\varphi_{m}a_{y}+\cos\theta a_{z}$$

(9) $$a_{m}\cdot a_{r}=\sin\theta \cos\left(\varphi -\varphi_{m}\right),\varphi_{m}=\frac{2\pi }{M}\left(m-1\right).$$

Assuming that the number of navigation signals in the airspace is L and the number of directional interference signals is *K*, jk represents the interfering signal, and the array signal vector of *UCA* can be expressed as

(10) $$x\left(t\right)=\sum_{l=1}^{L}p_{s_{l}}s_{l}\left(t\right)a_{s_{l}}\left(\theta_{l},\varphi_{l}\right)+\sum_{k=1}^{K}p_{jk}J_{k}\left(t\right)a_{j_{k}}\left(\theta_{k},\varphi_{k}\right)+\sqrt{p}n\left(t\right).$$

The direction vector $A_{UCA}$ of the whole array can be expressed as

(11) $$\begin{array}{c} A_{UCA}=\left[a_{s_{1}},a_{s_{2}},\cdots ,a_{s_{l}},a_{j_{1}},a_{j_{2}},\cdots ,a_{j_{k}}\right]\\ \left(\begin{array}{ccc} e^{j\varphi_{s_{1}}\left(m=1\right)} & \ldots & e^{j\varphi_{s_{l}}\left(m=1\right)}\\ \vdots & \ddots & \vdots \\ e^{j\varphi_{s_{1}}\left(m=M\right)} & \cdots & e^{j\varphi_{s_{l}}\left(m=1\right)} \end{array}\ddots \begin{array}{ccc} e^{j\varphi_{j_{1}}\left(m=1\right)} & \ldots & e^{j\varphi_{j_{k}}\left(m=1\right)}\\ \vdots & \ddots & \vdots \\ e^{j\varphi_{j_{1}}\left(m=M\right)} & \cdots & e^{j\varphi_{j_{k}}\left(m=M\right)} \end{array}\right) \end{array}.$$

Part of the expression in Formula (11) is shown as

(12) $$\left\{\begin{array}{c} \varphi_{s_{l}}=\frac{2\pi R}{\lambda }\sin\theta_{s_{l}}\cos\left(\phi_{s_{l}}-\phi_{m}\right)\\ \varphi_{j_{k}}=\frac{2\pi R}{\lambda }\sin\theta_{j_{k}}\cos\left(\phi_{j_{k}}-\phi_{m}\right)\\ \phi_{m}=\frac{2\pi }{M}\left(m-1\right) \end{array}\right.,\begin{array}{c} l=1,2,\cdots ,L\\ k=1,2,\cdots K\\ m=1,2,\cdots ,M \end{array}.$$

The direction of each signal arriving at the array is determined, so the direction vector of the antenna has also been determined, and the array signal weight vector,

(13) $$w={\left[w_{1},w_{2},\cdots ,w_{N}\right]}^{T},$$

becomes the optimization objective. The output of the array antenna system after the weighted operation is

(14) $$y\left(t\right)=w^{H}x\left(t\right).$$

## 3. Uniform Circular Array Anti-Interference Resolution

### 3.1. Concept

In the common concept of spatial immunity, the focus is on suppressing interference in a certain direction by generating null depth. The immunity degree of freedom refers to the maximum number of zeros that can be formed by the antenna direction map of this array at the same time; an M-element array can form zeros with a degree of freedom M−1 . The premise is that N interfering signal vectors in space are independent of each other, i.e., their direction vectors are linearly uncorrelated. In practical application, it is found that when two mutually independent interference sources come from a similar angle, the array antenna can only form an effective zero point at that angle, and at this time, an anti-interference degree of freedom can suppress multiple interference. Through this analysis, it can be seen that the bandwidths of different interference signals, such as time extension, signal correlation, filter order, and number of frequency points, have an impact on the degree of freedom. Hence, we analyze the anti-jamming resolution from the spatial spacing of the location of the interference source and the number of interference sources and give the prerequisite for the definition of the degree of freedom of the anti-jamming of the airspace domain.

**Figure 2:**
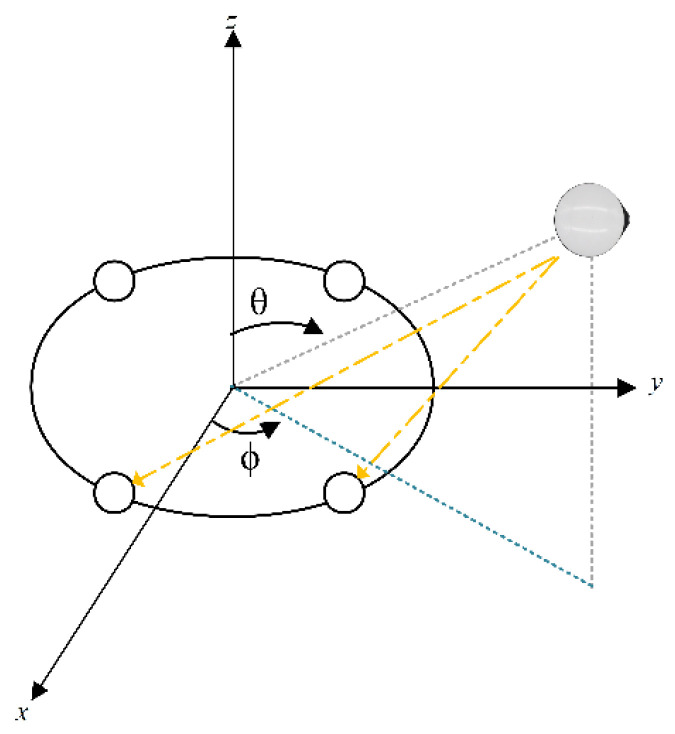
Single interference signal (The pointing of the interfering signal to each array element of the receiving antenna is the yellow dashed line, and the lines of other colors are auxiliary lines).

As shown in Figure 2, for a four-element uniform circular array schematic, assuming that the angular difference between the interfering signal and the receiving antenna array element is θ , the phase difference $\Delta_{\omega }$ between the arriving array element m and the array element m+1 with incident signal pitch angle θ and azimuthal angle θ is

(15) $$\Delta \omega =\frac{4\pi R}{\lambda }\sin\theta \sin\frac{\pi }{M}\sin\left[\frac{\pi }{M}\left(2k-1\right)-\varphi \right],k=1,2,\cdots ,M-1,$$

and the delay between the arrival of the signal at each array element is

(16) $$\tau_{k}=\frac{2\pi R}{\lambda }\cos\theta \cos\left[\frac{2\pi }{M}\left(k-1\right)-\varphi \right],k=1,2,\cdots ,M-1.$$

From Formula (15), it can be seen that the change rule of the phase difference satisfies the sinusoidal characteristic, which can be equated to a sinusoidal curve, with angular frequency $\frac{\pi }{M}$ , initial phase −φ , amplitude $\frac{4\pi R}{\lambda }\sin\theta \sin\frac{\pi }{M}$ , and sampling frequency 2k−1 . Similarly, it can be seen that array elements have a phase difference compared with the previous array element antenna, which will eventually form a receiving array with phase $\left[0,\Delta \omega ,2\Delta \omega ,3\Delta \omega \right]$ . From the abovementioned analysis, it can be seen that increasing the number of receiving antenna array elements can effectively improve the antenna resolution. Increasing the number of transmitting antennas can also achieve this effect.

**Figure 3:**
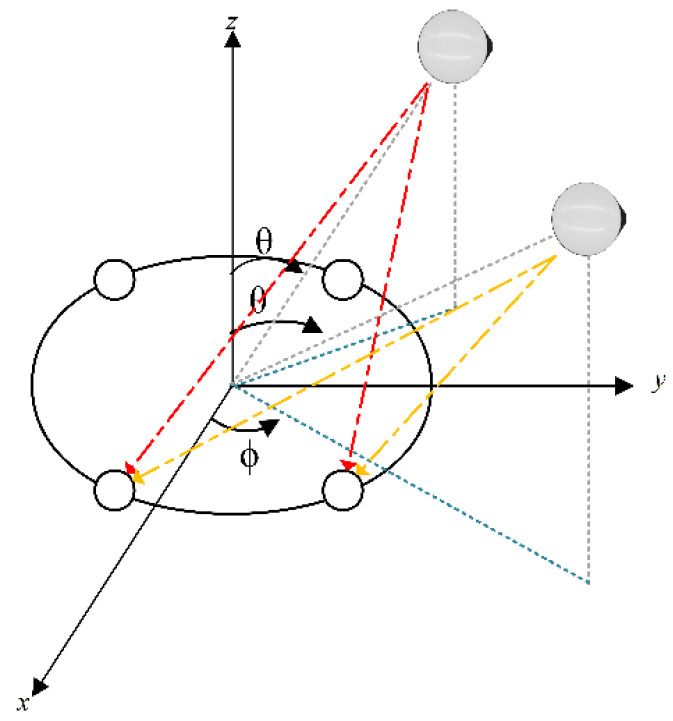
Multiple interference signals (The yellow and red dashed lines are the direction of the interfering signal pointing towards each array element of the receiving antenna, and the other colored lines are auxiliary lines.).

As shown in Figure 3, the transmitting antenna $J_{2}$ is added to Figure 2. Transmitting antennas $J_{1}$ and $J_{2}$ have the same pitch angle θ and respective azimuth angles of $\varphi_{1}$ and $\varphi_{2}$ , where $\varphi_{2}-\varphi_{1}=\Delta \omega$ . The receiving antenna receives the transmitting antenna $J_{2}$ signal phase with a phase difference of Δω that of the $J_{1}$ , and therefore the signal phase of $J_{2}$ is formed at the receiving antenna at each of its array elements The phase difference is $\left[4\Delta \omega ,5\Delta \omega ,6\Delta \omega ,7\Delta \omega \right]$ , so the phase of the signal formed at the receiving antenna by $J_{1}$ and $J_{2}$ should be $\left[0,\Delta \omega ,2\Delta \omega ,3\Delta \omega ,4\Delta \omega ,5\Delta \omega ,6\Delta \omega ,7\Delta \omega \right]$ , and the effect is analogous to that of generating eight virtual receiving antennas. When the number of array elements increases, its resolution increases synchronously; it can be seen that increasing the number of transmitting antennas K and receiving antenna array elements M can improve the azimuthal resolution of the receiving antenna.

The resolution of an interfering signal in an array direction is directly related to the rate of change of the array direction vector in the vicinity of the incoming direction. In the vicinity of the direction where the incoming direction vector of the interfering signal changes more rapidly, the snapshot data increase synchronously with the rate of change of the direction vector of the interfering signal, and the corresponding resolution also increases. The quantity representing the resolution is introduced as

(17) $$D\left(\theta ,\varphi \right)=\left\|\frac{da\left(\theta ,\varphi \right)}{d\theta d\varphi }\right\|\propto \left\|\frac{d\tau }{d\theta d\varphi }\right\|,$$

which indicates that as θ and φ increase, the resolution increases accordingly. Bringing Formula (16) into Formula (17) yields output

(18) $$D\left(\theta ,\varphi \right)\propto \left\|\frac{d\tau }{d\theta d\varphi }\right\|=\frac{2\pi R}{\lambda }\sin\varphi \sqrt{\frac{M}{2}},$$

illustrating that the resolution of the uniform circular array is a function of the azimuth, pitch angle, and number of array elements of the interfering signal, and as the direction of the interfering signal changes, so does the resolution, which is approximated by a sinusoidal function of the distribution, whose resolution is highest when the pitch angle is 90°.

### 3.2. Simulation Verification of Section 3.1

#### 3.2.1. Simulation Scenario 1

The theoretical analysis in Section 3.1 is verified by simulation, with parameters as shown in Table 2. Among them, 1575.42 MHz is the center frequency point of signal L1 of the GPS system. To facilitate the subsequent analysis, it is ensured that the pitch angle of the interference incidence is unchanged, and the azimuth angle varies from 0° to 180°.

**Figure 4:**
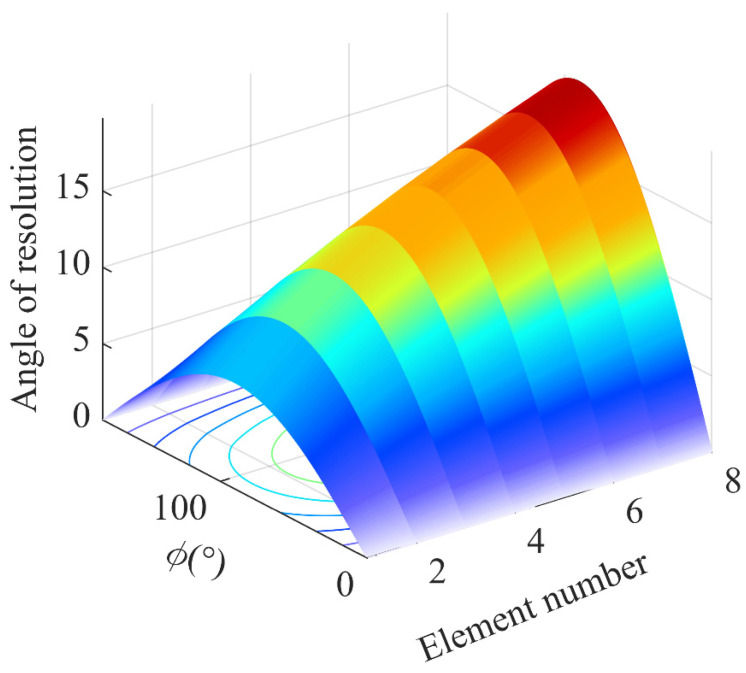
Effect of array element number on anti-jamming resolution.

The simulation results are shown in Figure 4. For UCA, the anti-jamming resolution is a function related to the number of arrays and the azimuth angle. In the case of a fixed azimuth, the four-array resolution is about 15° as the array number increases. Because of the single conditions of the abovementioned simulation, the calculation is made only from the angle of the receiving antenna; we do not consider the coupling between different interference signals in the airspace or the effect of signal pointing. The theory of Section 3.1 is simulated by combining the incidence angle of the interference signal in space and the anti-interference processing state of the receiving antenna.

#### 3.2.2. Simulation Scenario 2

Keeping the simulation conditions unchanged, two interference signals are added to the signal processing of the receiving antenna, and the simulation parameters are set as follows.

**Figure 5:**
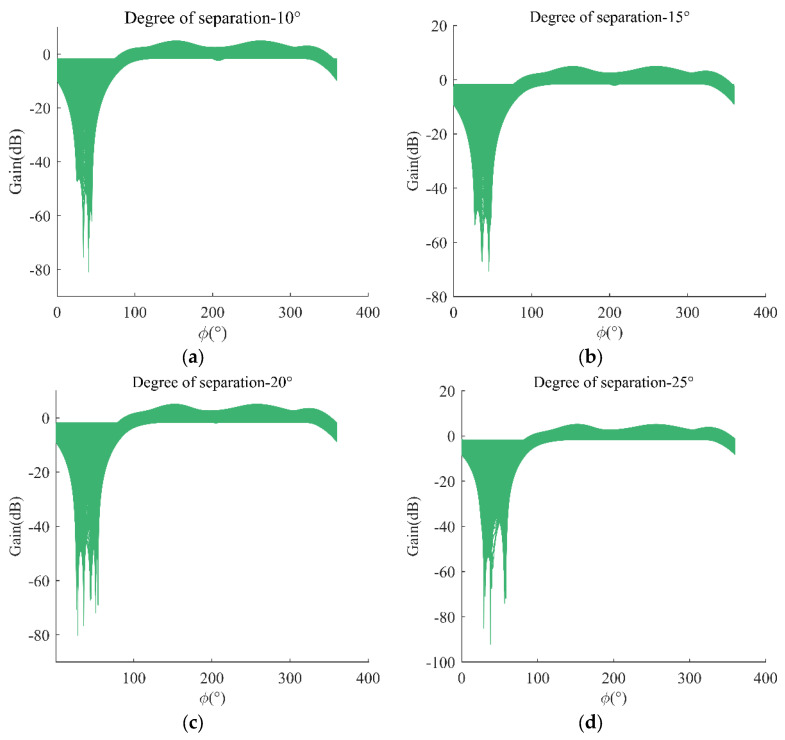
Relationship between azimuthal spacing of interfering signals and anti-interference resolution: (**a**) indistinguishable when azimuth is less than 15°; (**b**) critical state at azimuth equal to 15°; (**c**) resolution effect when azimuth angle is greater than 15°; (**d**) significant resolution at azimuths greater than 15°.

The number of array elements of the *UCA* is taken as four, and the azimuthal spacing of the interfering signal is taken as half the wavelength. With azimuthal spacing of the interfering signals of 10°, 15°, 20°, and 25°, the simulation results are shown in Figure 5.

From Figure 5, it can be seen that the array antenna cannot discriminate between two interfering signals when their spatial separation azimuths are less than or equal to 15° and can discriminate between them when the azimuths are greater than 15°, which further verifies the conclusions in Section 3.1. Analyzed in conjunction with the airspace filtering anti-interference algorithm, a single null depth can suppress multiple interferences when the azimuthal interval of the interfering signals is small. When the interval is large enough, the influence between the null depths is negligible. When the azimuthal interval of the interfering signals is at an angle at which the interfering signals are coupled to each other, the number of required null depths changes depending on the degree of coupling. When the interval is less than or equal to the resolution of the array antenna, it wastes spatial interference degrees of freedom.

Therefore, the anti-jamming degree of freedom of M−1 presupposes that the azimuthal distribution of the spatial jamming signal is larger than the resolution of the array antenna anti-jamming. However, the distance of the interference release, bandwidth of the interfering signal, and size of the interfering transmitting antenna should also be considered in practical applications.

## 4. Efficacy Analysis of Interference with Different Degrees of Freedom

### 4.1. Adaptive Array Criteria and Power Inversion Algorithms

The core problem of an adaptive array is the effective reception of useful signals, which is realized by adjusting the weight of each array element. These form the array weight vector, which directly determines the direction map of the adaptive array, i.e., the reception effect of the useful signal. This has two aspects: to align the main flap of the array direction map with the desired signal direction and to effectively suppress interference. To find the adaptive weight vector is a multi-parameter optimization problem under the criteria of the Minimum Mean Square Error (MMSE), Maximum Signal-to-interference Noise Ratio (MSINR), and Minimum Noise Variance (MNV), which are equivalent in an ideal case.

We base the analysis on the principle of the power inversion algorithm, and according to Formula (14), the average power output from the beam pointing formed by N snapshots is obtained from

(19) $$\begin{array}{c} p\left(w\right)=\frac{1}{N}\sum_{i=1}^{N}{\left|y\left(t\right)\right|}^{2}=\frac{1}{N}\sum_{i=1}^{N}{\left|w^{H}x\left(t\right)\right|}^{2}\\ =\sum_{l=1}^{L}\left[\frac{1}{N}\sum_{i=1}^{N}{\left|s\left(t\right)\right|}^{2}\right]{\left|w^{H}a_{s_{l}}\left(\theta_{l},\varphi_{l}\right)\right|}^{2}\\ +\sum_{k=1}^{K}\left[\frac{1}{N}\sum_{i=1}^{N}{\left|J\left(t\right)\right|}^{2}\right]{\left|w^{H}a_{j_{k}}\left(\theta_{k},\varphi_{k}\right)\right|}^{2}\\ +\frac{1}{N}{\left\|w\right\|}^{2}\sum_{i=1}^{N}{\left\|n\left(t\right)\right\|}^{2} \end{array},$$

which, when N→∞ , can be expressed as

(20) $$\begin{array}{c} p(w)=E\left[\left|y{\left(t\right)}^{2}\right|\right]=w^{H}E\left[x\left(t\right)x^{H}\left(t\right)\right]w=w^{H}R_{uu}w\\ \begin{array}{l} =\sum_{l=1}^{L}E\left[\left|s{\left(t\right)}^{2}\right|\right]{\left|w_{H}a_{S_{l}}\left(\theta_{l},\varphi_{l}\right)\right|}^{2}\\ +\sum_{l=1}^{L}E\left[\left|J{\left(t\right)}^{2}\right|\right]{\left|w_{H}a_{j_{k}}\left(\theta_{k},\varphi_{k}\right)\right|}^{2}\\ +\sigma_{n}^{2}{\left\|w\right\|}^{2} \end{array} \end{array},$$

where $R_{uu}$ is the covariance matrix of the array output power. To ensure accurate reception of the desired signals, complete suppression of interfering signals, and minimum array output power, the abovementioned problem can be formulated as an optimization problem:

(21) $$\left\{\begin{array}{c} \underset{w}{\min}E\left[\left|y{\left(t\right)}^{2}\right|\right]=\underset{w}{\min}w^{H}{\hat{R}}_{uu}w\\ s.t.w^{H}a_{j_{k}}\left(\theta_{k},\varphi_{k}\right)=0 \end{array}\right.,$$

Introducing a Lagrange multiplier solution gives the output of the array:

(22) $$w_{opt}=\frac{R_{uu}^{-1}a_{S_{l}}}{a_{S_{l}}^{H}R_{uu}^{-1}a_{S_{l}}}.$$

### 4.2. Interference Performance Evaluation Criteria

#### 4.2.1. Critical Power When the Number of Interfering Signals Is Less Than or Equal to Array Antenna Degrees of Freedom

The concept of the residual power of the interfering signal is introduced for the evaluation of the anti-interference performance of the array antenna. This represents the output power of the array after processing the interference, which is proportional to the null depth in the directional map formed by the anti-interference array. For GNSS receiver antennas, the purpose of anti-interference is to make the residual interference power zero while preserving the desired signal. The array output interference power is

(23) $$P_{j}=w^{H}R_{JJ}w,$$

where $R_{JJ}=\sum_{k=1}^{K}p_{j_{k}}a_{j_{k}}a_{j_{k}}^{H}$ is the covariance matrix of the interference signal. j represents the jamming signal, K represents the number of jamming signals, and JJ represents the ensemble of jamming signals. The array output power only represents the ability of the array to process the interfering signals. We introduce the Interference-to-Cancellation Ratio (*ICR*), which is a measure of the ability of an array antenna to process interfering signals. The specific value of *ICR* is denoted by $\frac{S}{J}$ . $P_{S}$ is the output power of the interference and noise after they have been processed by the array antenna, $P_{j}$ is the initial transmission power of the interference and noise, and JJ+NN denotes the ensemble of the interference and noise:

(24) $$\alpha_{\frac{S}{J}}=\frac{P_{S}}{P_{J}}=\frac{w_{S}^{H}R_{JJ+NN}w_{S}}{w_{J}^{H}R_{JJ+NN}w_{J}},$$

It can be seen that, when the array antenna can completely suppress the interfering signal at $\alpha_{\frac{S}{J}}>1$ , the interfering power does not exceed the immunity threshold, and when the array antenna fails to completely suppress the interfering signal at $\alpha_{\frac{S}{J}}<1$ , the interfering power exceeds it.

#### 4.2.2. Critical Power When Number of Interfering Signals Is Greater Than Array Antenna Degrees of Freedom

The *ICR* evaluation criterion satisfies the case where the number of interferences does not exceed the array degrees of freedom, but when super-DOF interferences are applied to the array antenna, the immunity degree of freedom M−1 is used to counteract J−M+1 interfering signals, at which point the immunity degree of freedom of the array antenna is fully consumed. At this time, the array anti-jamming algorithm cannot form an effective depth and number of zero traps on the interference power, and the power inversion algorithm cannot form a converged weights matrix. If the matrix is not the optimal weights matrix, we must look for the optimal weights matrix at the time $\alpha_{\frac{S}{J}}>1$ as a critical value parameter for the calculation of the interference power.

With the continuous change of the interference power, the Jamming Signal Ratio (*JSR*) also changes under the condition of maintaining the stability of the satellite signal. When the number of interferences exceeds the array antenna anti-jamming degrees of freedom, for $\alpha_{\frac{S}{J}}>1$ , based on the need to minimize the output power of the array, the model of the critical power of super-DOF interference is

(25) $$\left\{\begin{array}{c} \underset{JSR\to \infty }{\lim}\alpha_{\frac{S}{J}}=1\\ \min(w_{J}^{H}R_{JJ+NN}w_{J}) \end{array}\right..$$

where *JSR* is the jamming signal ratio and $\alpha_{\frac{S}{J}}$ is the *ICR*.

## 5. Simulation Analysis and Experimental Validation of Section 4

### 5.1. Simulation Analysis

#### 5.1.1. Simulation Parameter Settings

Typical scenarios of distributed multi-DOF jamming were simulated based on array antenna airspace anti-jamming performance and algorithmic analysis. We set the receiving antenna as a four-array element uniform circular array GNSS array antenna, and there was single-frequency interference. The simulation parameters are shown in Table 3, and 1575.42 MHz was one of the center frequencies of GNSS. The simulation results are generalized and consistent for other navigation systems. The azimuth and elevation angle settings of the interference signal are shown in Table 4.

#### 5.1.2. Simulation Scenario 3

We simulate and verify the theoretical analysis in Section 4.2.1. The anti-jamming degree of freedom of the four-element *UCA* antenna is M−1=3 , according to the power inversion algorithm, to form the corresponding zero-trapping in the direction of the interference to process the interference signal, to ensure that the array antenna outputs the correct directional map for the reception of satellite signals. In the airspace, no more than three mutually independent interference signals are gradually applied, and the ratio of the array output power before and after the anti-jamming of the array is calculated. We observe the change rule of the critical interference power value of the array when there are different numbers of interference signals in the airspace.

**Figure 6:**
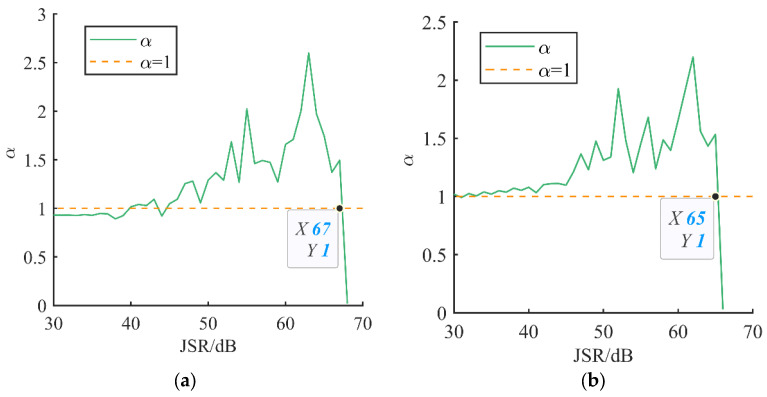
Value of *ICR* when JSR is increased. Number of interfering signals: (**a**) 2; (**b**) 3.

Figure 6a,b, respectively, show how the critical interference power value of the array changes under the premise that the number of interfering signals in the airspace does not exceed the array’s anti-jamming degrees of freedom; the associated values are shown in Table 5. When the number of interfering signals remains unchanged and the interfering power does not exceed the anti-jamming capability of the array antenna, the *ICR* value is greater than 1. When the interfering power is greater than the anti-jamming capability of the array antenna, the *ICR* value decreases drastically. When the number of interfering signals increases, the array antenna uses up more anti-jamming degrees of freedom, thus decreasing its ability to deal with interfering signals while also decreasing the required critical interfering signal power. The orange dashed line in the horizontal axis of the figure indicates that the value of the *ICR* is 1, which serves as a schematic line for judging the critical power of interference. The vertical axis indicates the value of the interference cancellation ratio under different values of *JSR*. As the *JSR* increases, the critical power is reached when the *ICR* shows a sharp decrease.

Since noise power is added in the calculation of *ICR*, the power of the interfering signal is calculated with the noise power as the benchmark, which can also be equated to the Signal-to-Interference-Plus-Noise Ratio (SINR) of the received signal of the array antenna. Under the condition of not exceeding the anti-interference freedom by increasing the number of interfering signals, as shown in Table 5, it is found that for each additional interfering signal, the critical interfering power decreases by 2–4 dB, with an average decrease of 3 dB.

**Figure 7:**
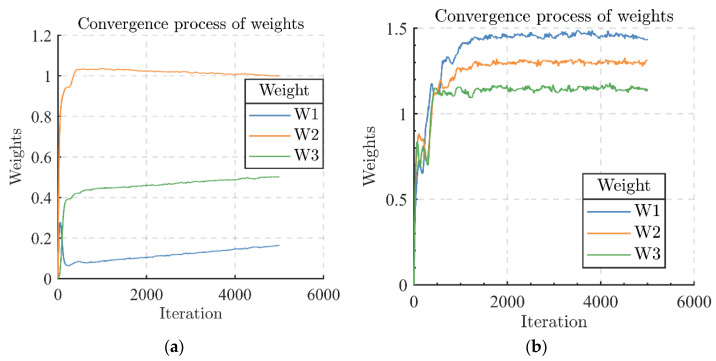
Convergence process of weights. Number of interfering signals: (**a**) 2; (**b**) 3.

Figure 7a,b, respectively, represent the convergence process of the array antenna weights when two and three mutually independent interference signals are applied in the airspace when the dry signal ratio *JSR* is kept constant. As the number of interfering signals increases, the convergence of the weights of the array antenna slows down. The number of iterations for the convergence of the weights increases from about 200 to about 1500, which can lead to an increase in the time needed for the positioning of the GNSS receiver by at least a factor of 5–10.

#### 5.1.3. Simulation Scenario 4

**Figure 8:**
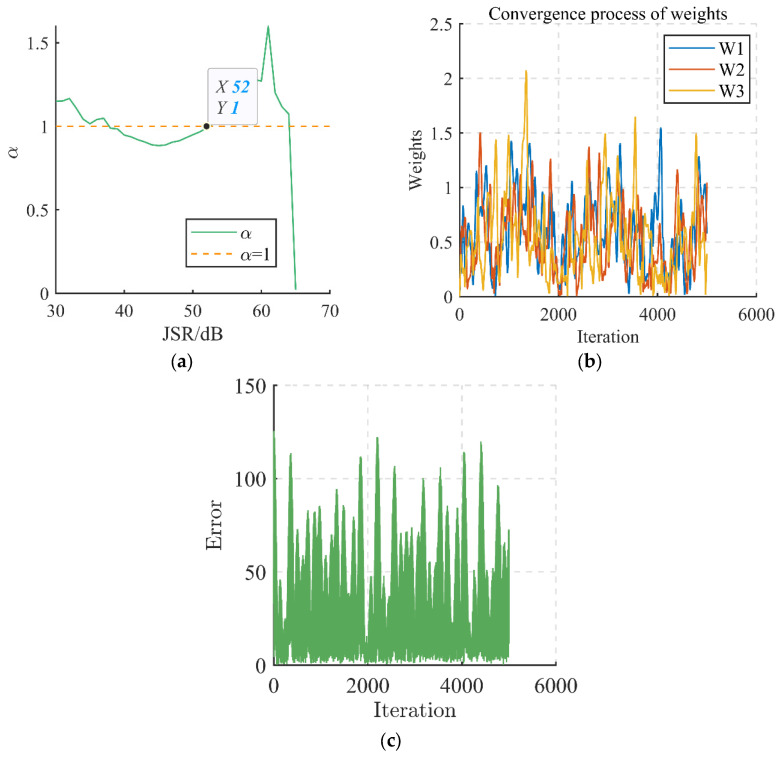
Super-degree-of-freedom interference characterization. (**a**) Value of *ICR* when *JSR* is increased; (**b**) convergence process of weights; (**c**) convergence process of error.

We simulate and verify the theoretical analysis in Section 4.2.2. Figure 8 shows the simulation results when the number of interfering signals exceeds the array antenna’s immunity degrees of freedom.

Figure 8a indicates that, as the JSR increases, the ICR appears critical at a JSR of 63 dB. According to the conclusion of simulation scenario 3, the critical interference power increases by about 3 dB when the number of array elements is doubled, which satisfies the conclusion of simulation scenario 3. According to the model in Section 4.2.2 and Figure 8b,c, it can be seen that the array weights and error signals do not converge when the *JSR* is 63 dB, and the output power of the array is not the minimum, which does not satisfy the super-DOF interference model. From Figure 8a, it can be seen that the array interference pair cancellation ratio is about one when the *JSR* is 52 dB, and the array output power is the minimum at this time. The array antenna arrives at the critical state of anti-jamming when the *JSR* is 52 dB, and the critical jamming power is −57 dBm at this time.

### 5.2. Experimental Verification

#### 5.2.1. Equipment Installation

**Figure 9:**
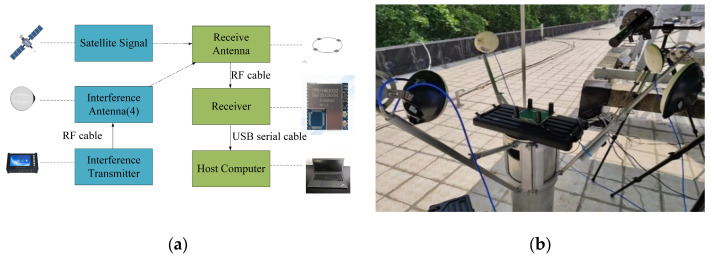
Test scenarios. (**a**) Link diagram; (**b**) test environment.

A test environment of spatial multi-DOF interference was constructed for the simulation analysis in Section 5.1, and the critical interference power under different interference degrees of freedom was verified. The test used four omnidirectional transmitter antennas, a four-array antenna against interference, and a receiver module. The interference signal generator was used to simulate four independent and different interference signals, and the power of the transmitter side of the signal simulator was adjustable. The localization information solved by the receiver module was read by the host computer. During the test, the localization information of the current position was lost according to the software of the upper computer as the criterion for determining critical power. The test schematic is shown in Figure 9a.

#### 5.2.2. Comparison of Simulation and Test Results

**Figure 10:**
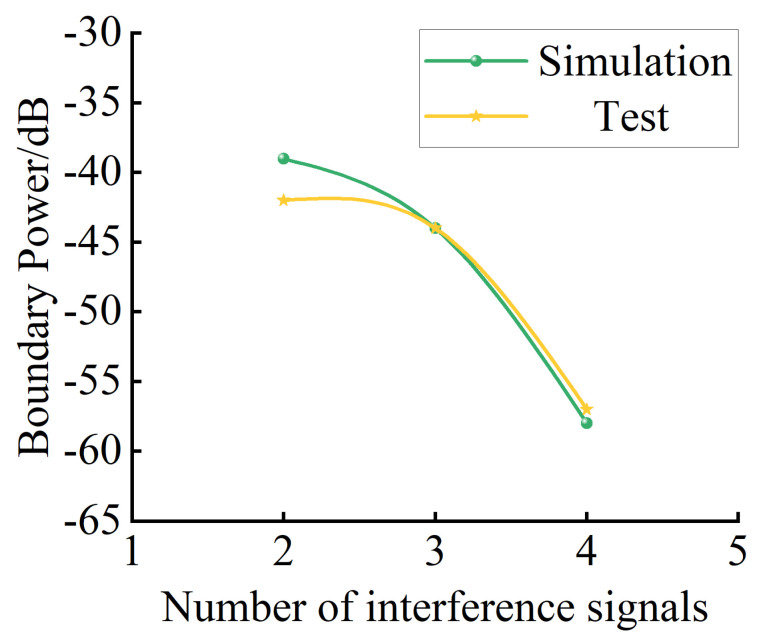
Comparison of results.

Figure 10 shows the critical power change rule of array antenna interference with different numbers of interfering signals, and the experimental and simulation results are consistent. When the number of interfering signals does not exceed the array antenna’s anti-jamming degrees of freedom, the critical interference power decreases by about 3 dB for each additional interfering signal in the airspace. When the number of interfering signals exceeds the array antenna’s anti-jamming degrees of freedom, the critical interference power decreases by about 15 dB for each additional signal. According to the experimental results, when a distributed jamming source is used to interfere with GNSS array antennas in practice, super-DOF interference can achieve better results. Due to the interference of other signals and uncertain space and cable insertion loss in the airspace during the experiment, there was an acceptable error of ±5 dB between the experimental and simulation results.

## 6. Conclusions

We proposed prerequisites for multi-DOF interference with GNSS array antennas and investigated the change rule of critical interference power under different numbers of interfering signals. Our main contributions are as follows:

(1) Assuming that the interfering signals are independent of each other, the azimuthal interval of the interfering signals must be greater than 15° for multi-DOF jamming of a four-array element UCA antenna;
(2) The four-array UCA antenna’s weight array and signal error do not converge during super-DOF jamming, and the jamming signal cannot be processed effectively;
(3) For four-array UCA interference, when the number of interfering signals does not exceed the anti-jamming degrees of freedom, the critical interference power decreases by about 3 dB for each additional interfering signal, and the critical interference power decreases by about 15 dB in the case of super-DOF interference.

## 7. Discussion

We gave the multi-critical interference power for a typical GNSS antenna array and modeled the super-DOF critical interference power. However, there are several issues to be considered: (1) the proposed precondition of multi-DOF interference is ideal and must be combined with variables such as the actual size of the antenna in practical applications; (2) the airspace link attenuation in the test was calculated and would be affected by other uncertainties, leading to changes in the error; and (3) Super-DOF jamming must consume more resources, and the same effect is considered achievable through jamming integration techniques.

## Figures and Tables

**Table 1:** Frequently used symbols.

Symbol	Explanation
${\left[\cdot \right]}^{H}$	Hermitian transpose
${\left[\cdot \right]}^{T}$	Transpose
$\left\|\cdot \right\|$	Norm of vector
j	Imaginary unit

**Table 2:** Simulation parameters.

Parameter	Value
Light speed	$3\times 10^{8}$ m/s
Carrier frequency	1575.42 MHz
Array element spacing	1/2 wavelength
Interference power	−76 dBm
Number of elements	1–8
Range of azimuth angles	0–180°

**Table 3:** Simulation parameters.

Parameter	Value
Satellite signal power	−130 dBm
Satellite signal angles in elevation	45°
Satellite signal angles in azimuth	120°
Array geometry	*UCA*
Number of interferences	1–4
Interference type	single-frequency interference
Azimuth of interference signal distribution	0–360°, uniform distribution

**Table 4:** Azimuth and elevation of interference signals.

Signal	Azimuth	Elevation
Interference 1	120°	60°
Interference 2	180°	50°
Interference 3	90°	45°
Interference 4	0°	60°

**Table 5:** Critical interference power.

Number of Interference Signals	${JSR}_{\mathrm{boundary}}$	$P_{Jammer}$
2	67 dB	−42 dBm
3	65 dB	−44 dBm

## Data Availability

The data presented in this study are available on request from the corresponding author.
